# Probing cerebellar involvement in cognition through a meta-analysis of TMS evidence

**DOI:** 10.1038/s41598-021-94051-5

**Published:** 2021-07-20

**Authors:** Daniele Gatti, Luca Rinaldi, Ioana Cristea, Tomaso Vecchi

**Affiliations:** 1 Department of Brain and Behavioral Sciences, University of Pavia, Piazza Botta 6, 27100 Pavia, Italy; 2 Cognitive Psychology Unit, IRCCS Mondino Foundation, Pavia, Italy

**Keywords:** Cognitive neuroscience, Human behaviour

## Abstract

Traditionally, the cerebellum has been linked to motor coordination, but growing evidence points to its involvement in a wide range of non-motor functions. Though the number of studies using transcranial magnetic stimulation (TMS) to investigate cerebellar involvement in cognitive processes is growing exponentially, these findings have not yet been synthesized in a meta-analysis. Here, we used meta-analysis to estimate the effects of cerebellar TMS on performance in cognitive tasks for healthy participants. Outcomes included participants’ accuracy and response times (RTs) of several non-motor tasks performed either during or after the administration of TMS. We included overall 41 studies, of which 44 single experiments reported effects on accuracy and 41 on response times (RTs). The meta-analyses showed medium effect sizes (for accuracy: *d* = 0.61 [95% *CI* = 0.48, .073]; for RTs: *d* = 0.40 [95% *CI* = 0.30, 0.49]), with leave-one-out analyses indicating that cumulative effects were robust, and with moderate heterogeneity. For both accuracy and RTs, the effect of TMS was moderated by the stimulation paradigm adopted but not by the cognitive function investigated, while the timing of the stimulation moderated only the effects on RTs. Further analyses on lateralization revealed no moderation effects of the TMS site. Taken together, these findings indicate that TMS administered over the cerebellum is able to modulate cognitive performance, affecting accuracy or RTs, and suggest that the various stimulation paradigms play a key role in determining the efficacy of cerebellar TMS.

## Introduction

The human cerebellum has been traditionally studied in relation to motor functions. Yet growing evidence supports the involvement of the cerebellum in a wide range of non-motor functions, spanning from the cognitive to the emotional domains (e.g., emotive processing^1^; perceptual processing^2^; cognitive processing^3^; language^4^; and for an overall overview see^5,6^). To account for the cerebellar contribution in non-motor processes, Schmahmann^7^ proposed the so-called *dysmetria of thought* hypothesis*,* arguing that the cerebellum performs the same computational processes across all domains in which it is involved in. Evidence supporting this perspective comes from studies showing that the microstructure of the cerebellar cortex is uniform^8^ and that cerebro-cerebellar connections are segregated^9–11^. Specifically, structural uniformity would underlie functional uniformity and the segregated cerebro-cerebellar connections would allow specific cerebellar modules to participate in specific cognitive functions^12^.

Cerebellar modulation of motor and non-motor behavior has been primarily investigated through neurostimulation techniques, such as transcranial direct current stimulation (tDCS) or transcranial magnetic stimulation (TMS). This growing evidence was synthesized in several qualitative reviews^13–16^ as well as in a recent meta-analysis, demonstrating that anodal and cathodal cerebellar tDCS are effective in modulating participants’ performance (i.e., whether in the form of cognitive impairment or enhancement) and that the effect of cerebellar tDCS on motor functions is higher compared with non-motor functions^17^. Yet, no systematic review has insofar examined cerebellar modulation by synthesizing the evidence for TMS.

Here, we are therefore interested in quantifying the effect of TMS across non-motor functions. This choice is motivated by the significant increase of TMS studies investigating cerebellar involvement in non-motor functions, as well as by the theoretical difficulty to frame cerebellar involvement in cognitive processing. Furthermore, we note that the inclusion of the studies investigating motor functions could be problematic from a metanalytic point of view as they are highly heterogeneous: that is, the studies that investigated cerebellar involvement in motor processes using TMS highly differ in terms of paradigms adopted^18–24^, dependent variables used^25–27^ and population tested^28,29^, and could not consequently be directly comparable.

TMS is a noninvasive brain stimulation technique that uses electromagnetic induction principles to induce electrical currents in the brain^30,31^. Albeit the precise mechanisms through which TMS influences brain function are currently not fully understood, this technique is thought to stimulate axons placed in the cortex or in the white matter and to not directly modulate cell bodies activity^32^. Typically, TMS is used to investigate the link between the activity of a certain brain area and a motor or non-motor function, since a change in behavior induced by TMS (i.e., generally measured using accuracy or response times, RTs) is causally informative of the relationship among the area stimulated and the function investigated^33^. It should be noted that TMS does not necessarily cause performance disruption; in some specific conditions, TMS can also induce performance enhancement. Performance enhancement has been ascribed to direct modulation of a cortical region involved in one function, but also to indirect modulation (i.e., diaschisis), to non-specific effect of the stimulation (e.g., intersensory facilitation), or to addition-by-subtraction processes, which is a disruption of those processes supposed to compete or distract from task performance^34^. Within this context is should be noted that, given the assumption that every performance modulation (impairment or enhancement) is thought to be caused by the stimulation, it is impossible to observe “real” negative effects. This point was indeed handled by previous meta-analyses by computing both absolute and signed effect sizes, but focusing mainly on the former^17^.

TMS has been employed to the study of cerebellar functions, both in the motor and non-motor domains^35^. Cerebellar TMS aims at investigating temporal features of cerebellar-cortical connectivity^36^ as well as more basic features of cerebellar involvement in various processes. For instance, for the motor domain, TMS evidence indicates that the cerebellum exerts an inhibitory effect on motor areas^18^, that it is involved in saccadic adaptation^19,20^, and in the acquisition and extinction of conditioned responses^21–24^. Similarly, for the cognitive domain, TMS evidence supports cerebellar involvement in semantic memory^37^, working memory^38^, executive functions^39^, social cognition^40^ and spatial processing^41^. However, the cerebellar cortex is significantly different compared with other cortical areas, such as the frontal or temporal regions of the brain, in terms of number of neurons, composition of neurons and glia and their organization^8^. Because of these key differences, it is not known whether the effects of TMS could be similar to the effects reported for the other areas of the cerebral cortex.

Here, we therefore propose a systematic review and meta-analysis to quantify the effect of TMS in non-motor, cognitive domains. Despite the proliferation of this literature, no such review currently exists. We focus on studies of non-motor function because as a whole they are more homogeneous in terms of paradigms, dependent variables and populations. We also aim to parse variability by looking at potential moderators of effect, such as differences in stimulation paradigms in terms of burst pattern (e.g., double pulse, triple pulse, etc.) or timing (online vs. offline), function investigated, or cerebellar site stimulated. Quantifying the effect of TMS across non-motor functions or possible moderators identified could provide parameters for the use by future TMS studies targeting the cerebellum. As any performance modulation (impairment or enhancement) could be potentially ascribed to TMS influence, in line with previous methodological approaches^17^ we ran different meta-analyses on absolute and signed effect sizes: the former approach indeed accounts for any cognitive modulation induced by cerebellar TMS (whether in the form of impairment or enhancement), while the latter may be useful to gain insights into the specific direction of the effect (whether cerebellar TMS typically leads to cognitive impairment or enhancement).

## Materials and methods

### Identification and selection of studies

To identify potential studies for inclusion in the meta-analysis, we systematically searched Pubmed for studies conducted from January 2000 (the first TMS study targeting the cerebellum and investigating non-motor functions has been performed by Rami et al.^42^) to January 2021. We used the following search string: “((((((transcranial magnetic stimulation) OR tms) OR rtms) OR theta burst) OR tbs)) AND ((cerebellum) OR cerebellar)”. We also manually checked references for narrative reviews investigating cerebellar involvement in cognition using neurostimulation techniques^13–16^. Study identification and selection was performed by DG.

We included studies with the following characteristics: (i) a sample composed by healthy and adult participants, (ii) the administration of TMS for at least one cerebellar site, (iii) the presence of a cognitive (i.e., non-motor) performance index, (iv) the use of accuracy and/or response times (RTs) as dependent variables, (v) the adoption of cerebellar TMS protocols with the explicit intention to modulate cerebellar function.

From each study, we extracted: the sample size, the dependent variable(s) of interest (accuracy, response times), the cerebellar site stimulated, the stimulation paradigm adopted (e.g., theta-burst stimulation, triple-pulse TMS, single-pulse TMS), the design of the study, the control condition adopted, the timing of the stimulation, the mean and standard deviation of participants’ performance in the various conditions, and the specific cognitive function investigated.

### Effect size calculations

Accuracy and RTs were the dependent variables of interest. For each dependent variable, from each study, we included only one effect size (see Supplementary Material for more information regarding which effect was chosen for each study). This procedure is considered as the most straightforward one in case of within-participants dependencies in the same study^43^.

The effect size used was Cohen’s *d*^44^. Cohen’s *d* for between-participants designs is defined as the mean standardized difference between the two measurements (in our case, cerebellar TMS vs. control area / no TMS / sham stimulation). For within-participants designs, Cohen’s *d* computation requires taking into account the correlation between the two measurements (cerebellar TMS vs. control area / sham stimulation / no TMS; see Table 1 for more information regarding the control condition used by each study included); that is, the mean difference between the two measurements is divided by:

**Table 1:** The studies included in this meta-analysis.

Study ID	Function	Paradigm	Timing	Design	Dep.var	Exp	N	TMS site	CC	ES.5	V.5	ES.75	V.75	Agg	ES.5 direction	ES.75 direction
^37^	Semantic Memory	cTBS	Offline	Between	RTs	1	24	RC	No TMS	0,83	0,18	0,83	0,18	N	0,83	0,83
^37^	Semantic Memory	cTBS	Offline	Between	RTs	1	22	MC	No TMS	0,24	0,18	0,24	0,18	N	-0,24	-0,24
^38^	Working Memory	spTMS	Online	Within	Accuracy	1	17	RC	No TMS	0,12	0,06	0,16	0,03	N	0,12	0,16
^38^	Working Memory	spTMS	Online	Within	RTs	1	17	RC	No TMS	0,32	0,06	0,43	0,03	N	-0,32	-0,43
^39^	Executive Functions	cTBS	Offline	Between	Accuracy	1	27	RC vs LC	Cerebellum	0,79	0,16	0,79	0,16	N	NA	NA
^40^	Social Cognition	tpTMS	Online	Within	Accuracy	1	32	MC	Vertex	0,35	0,03	0,50	0,02	N	-0,35	-0,50
^40^	Social Cognition	tpTMS	Online	Within	RTs	1	32	MC	Vertex	0,10	0,03	0,14	0,02	N	0,10	0,14
^40^	Social Cognition	tpTMS	Online	Within	Accuracy	2	48	MC	Vertex	0,35	0,02	0,49	0,01	N	-0,35	-0,49
^40^	Social Cognition	tpTMS	Online	Within	RTs	2	48	MC	Vertex	0,03	0,02	0,04	0,01	N	-0,03	-0,04
^40^	Social Cognition	tpTMS	Online	Within	Accuracy	3	32	LC	Vertex	0,44	0,03	0,63	0,02	N	-0,44	-0,63
^40^	Social Cognition	tpTMS	Online	Within	RTs	3	32	LC	Vertex	0,09	0,03	0,13	0,02	N	0,09	0,13
^41^	Spatial cognition	tpTMS	Online	Within	Accuracy	1	12	MC	Sham	0,42	0,09	0,58	0,05	N	-0,42	-0,58
^41^	Spatial cognition	tpTMS	Online	Within	RTs	1	12	MC	Sham	0,04	0,08	0,05	0,04	N	-0,04	-0,05
^41^	Spatial cognition	tpTMS	Online	Within	Accuracy	2	12	LC	Sham	0,12	0,08	0,16	0,04	N	0,12	0,16
^41^	Spatial cognition	tpTMS	Online	Within	RTs	2	12	LC	Sham	0,06	0,08	0,09	0,04	N	0,06	0,09
^42^	Memory (various sub-types)	HFrTMS	Online	Within	Accuracy	1	16	RC	No TMS	0,40	0,04	0,56	0,02	N	NA	NA
^47^	Spatial cognition	spTMS	Online	Within	Accuracy	1	30	MC	Vertex	0,13	0,03	0,18	0,02	N	NA	NA
^47^	Spatial cognition	spTMS	Online	Within	RTs	1	30	MC	Vertex	0,02	0,03	0,03	0,02	N	NA	NA
^47^	Spatial cognition	spTMS	Online	Within	Accuracy	2	24	LC	Vertex	0,52	0,05	0,64	0,03	N	NA	NA
^47^	Spatial cognition	spTMS	Online	Within	RTs	2	24	LC	Vertex	0,53	0,05	0,71	0,02	N	NA	NA
^48^	Semantic Memory	tpTMS	Online	Within	Accuracy	1	24	RC	Vertex	0,50	0,05	0,62	0,02	N	-0,50	-0,62
^48^	Semantic Memory	tpTMS	Online	Within	RTs	1	24	RC	Vertex	0,26	0,04	0,36	0,02	N	-0,26	-0,36
^48^	Semantic Memory	tpTMS	Online	Within	Accuracy	2	20	RC	Visual C	0,76	0,06	0,90	0,03	N	-0,76	-0,90
^48^	Semantic Memory	tpTMS	Online	Within	RTs	2	20	RC	Visual C	0,13	0,05	0,18	0,02	N	-0,13	-0,18
^49^	Memory (various sub-types)	tpTMS	Online	Within	Accuracy	1	24	RC	Vertex	0,11	0,05	0,15	0,02	N	-0,11	-0,15
^49^	Memory (various sub-types)	tpTMS	Online	Within	Accuracy	2	32	RC	Vertex	0,36	0,03	0,51	0,02	N	-0,36	-0,51
^58^	Semantic Memory	cTBS	Offline	Within	RTs	1	19	LC vs RC	Cerebellum	0,54	0,06	0,77	0,03	N	NA	NA
^59^	Attention	cTBS	Offline	Between	Accuracy	1	45	RC vs LC & Sham	Sham & Cerebellum	0,96	0,11	0,96	0,11	N	NA	NA
^60^	Semantic Memory	cTBS	Offline	Within	Accuracy	1	4	MC vs RC	Cerebellum	0,92	0,36	1,24	0,18	N	NA	NA
^60^	Semantic Memory	cTBS	Offline	Within	RTs	1	8	MC vs RC	Cerebellum	0,96	0,18	1,09	0,09	N	NA	NA
^61^	Semantic Memory	cTBS	Offline	Between	RTs	1	24	MC vs RC	Cerebellum	0,66	0,18	0,66	0,18	N	NA	NA
^62^	Timing	LFrTMS	Offline	Between	Accuracy	1	26	RC	Sham	0,51	0,16	0,51	0,16	N	-0,51	-0,51
^63^	Working Memory	dpTMS	Online	Within	Accuracy	1	9	RC	No TMS	1,51	0,24	2,12	0,12	N	-1,51	-2,12
^63^	Working Memory	dpTMS	Online	Within	RTs	1	9	RC	No TMS	0,13	0,11	0,19	0,06	N	-0,13	-0,19
^64^	Episodic Memory	HFrTMS	Offline	Within	Accuracy	1	24	RC	Visual C	0,38	0,04	0,54	0,02	N	0,38	0,54
^65^	Attention	iTBS	Offline	Within	Accuracy	1	14	fmri_based	Cerebellum	0,13	0,07	0,19	0,04	N	NA	NA
^66^	Working Memory	tpTMS	Online	Within	Accuracy	1	18	RC	Vertex	0,60	0,07	0,83	0,03	N	-0,60	-0,83
^66^	Working Memory	tpTMS	Online	Within	RTs	1	18	RC	Vertex	0,15	0,06	0,21	0,03	N	-0,15	-0,21
^66^	Working Memory	tpTMS	Online	Within	Accuracy	2	18	RC	Vertex	0,42	0,06	0,55	0,03	N	-0,42	-0,55
^66^	Working Memory	tpTMS	Online	Within	RTs	2	18	RC	Vertex	0,10	0,06	0,14	0,03	N	-0,10	-0,14
^67^	Social Cognition	tpTMS	Online	Within	Accuracy	1	36	LC	Vertex	0,11	0,03	0,15	0,01	N	-0,11	-0,15
^67^	Social Cognition	tpTMS	Online	Within	RTs	1	36	LC	Vertex	0,06	0,03	0,08	0,01	N	-0,06	-0,08
^67^	Social Cognition	tpTMS	Online	Within	Accuracy	2	20	LC	Visual C	0,17	0,05	0,24	0,03	N	-0,17	-0,24
^67^	Social Cognition	tpTMS	Online	Within	RTs	2	20	LC	Visual C	0,38	0,05	0,54	0,03	N	0,38	0,54
^67^	Social Cognition	tpTMS	Online	Within	Accuracy	3	20	LC	Visual C	0,18	0,05	0,25	0,03	N	-0,18	-0,25
^67^	Social Cognition	tpTMS	Online	Within	RTs	3	20	LC	Visual C	0,47	0,06	0,66	0,03	N	-0,47	-0,66
^68^	Social Cognition	tpTMS	Online	Within	Accuracy	1	20	LC	Vertex	0,49	0,06	0,69	0,03	N	-0,49	-0,69
^68^	Social Cognition	tpTMS	Online	Within	RTs	1	20	LC	Vertex	0,11	0,05	0,15	0,03	N	0,11	0,15
^68^	Social Cognition	tpTMS	Online	Within	Accuracy	2	20	LC	Vertex	0,69	0,06	0,92	0,03	N	-0,69	-0,92
^68^	Social Cognition	tpTMS	Online	Within	RTs	2	20	LC	Vertex	0,05	0,05	0,07	0,03	N	0,05	0,07
^69^	Timing	LFrTMS	Offline	Within	Accuracy	1	10	RC vs LC	Cerebellum	0,71	0,12	0,94	0,06	N	NA	NA
^70^	Social Cognition	tpTMS	Online	Between	Accuracy	1	40	RC	No TMS	0,04	0,10	0,04	0,10	N	NA	NA
^70^	Social Cognition	tpTMS	Online	Between	RTs	1	40	RC	No TMS	0,47	0,10	0,47	0,10	N	NA	NA
^71^	Semantic Memory	cTBS	Offline	Within	RTs	1	21	RC	Vertex	0,56	0,06	0,78	0,03	N	0,56	0,78
^71^	Semantic Memory	cTBS	Offline	Within	RTs	2	20	LC	Vertex	0,29	0,05	0,40	0,03	N	-0,29	-0,40
^72^	Timing	HFrTMS	Online	Within	RTs	1	16	RC	No TMS	0,49	0,07	0,69	0,04	N	0,49	0,69
^73^	Timing	cTBS	Offline	Between	Accuracy	1	24	MC	Sham	1,00	0,19	1,00	0,19	N	-1,00	-1,00
^74^	Timing	LFrTMS	Offline	Within	Accuracy	1	9	LC	DLPFC	0,31	0,12	0,44	0,06	N	-0,31	-0,44
^74^	Timing	HFrTMS	Online	Within	Accuracy	2	8	LC	Vertex	0,04	0,13	0,06	0,06	N	0,04	0,06
^75^	Music	LFrTMS	Offline	Within	RTs	1	14	RC	Sham	0,56	0,08	0,78	0,04	N	-0,56	-0,78
^76^	Semantic Memory	LFrTMS	Offline	Between	RTs	1	43	RC	Vertex	0,69	0,10	0,69	0,10	N	-0,69	-0,69
^77^	Timing	cTBS	Offline	Within	Accuracy	1	14	RC	DLPFC	0,44	0,08	0,62	0,04	N	-0,44	-0,62
^78^	Spatial cognition	LFrTMS	Offline	Within	Accuracy	1	8	LC	Neck	2,15	0,83	3,37	0,42	N	NA	NA
^79^	Semantic Memory	LFrTMS	Offline	Between	Accuracy	1	24	RC vs LC	Cerebellum	3,04	0,27	3,04	0,27	Y	NA	NA
^79^	Semantic Memory	LFrTMS	Offline	Between	RTs	1	24	RC vs LC	Cerebellum	0,69	0,13	0,69	0,13	Y	NA	NA
^80^	Executive Functions	cTBS	Offline	Between	Accuracy	1	14	LC	Sham	0,30	0,29	0,30	0,29	N	-0,30	-0,30
^80^	Executive Functions	cTBS	Offline	Between	RTs	1	14	LC	Sham	1,62	0,38	1,62	0,38	N	-1,62	-1,62
^80^	Executive Functions	cTBS	Offline	Between	Accuracy	2	14	LC	Sham	0,00	0,29	0,00	0,29	N	0,00	0,00
^80^	Executive Functions	cTBS	Offline	Between	RTs	2	14	LC	Sham	2,17	0,45	2,17	0,45	N	-2,17	-2,17
^81^	Executive Functions	cTBS	Offline	Between	Accuracy	1	28	LC	Sham	0,52	0,15	0,52	0,15	N	0,52	0,52
^81^	Executive Functions	cTBS	Offline	Between	RTs	1	28	LC	Sham	0,71	0,15	0,71	0,15	N	-0,71	-0,71
^81^	Executive Functions	cTBS	Offline	Between	Accuracy	2	28	LC	Sham	0,22	0,14	0,22	0,14	N	-0,22	-0,22
^81^	Executive Functions	cTBS	Offline	Between	RTs	2	28	LC	Sham	0,19	0,14	0,19	0,14	N	0,19	0,19
^82^	Executive Functions	cTBS	Offline	Within	RTs	1	12	LC	Sham	0,22	0,05	0,29	0,03	Y	NA	NA
^83^	Executive Functions	LFrTMS	Offline	Within	Accuracy	1	16	RC vs LC	Cerebellum	0,47	0,05	0,59	0,02	Y	NA	NA
^83^	Executive Functions	LFrTMS	Offline	Within	RTs	1	16	RC vs LC	Cerebellum	0,57	0,05	0,80	0,02	Y	NA	NA
^84^	Social Cognition	HFrTMS	Online	Within	RTs	1	15	MC	Sham	0,33	0,07	0,47	0,04	N	-0,33	-0,47
^85^	Working Memory	dpTMS	Online	Within	Accuracy	1	23	RC	No TMS	0,85	0,06	1,07	0,03	N	-0,85	-1,07
^85^	Working Memory	dpTMS	Online	Within	RTs	1	23	RC	No TMS	0,33	0,05	0,44	0,02	N	-0,33	-0,44
^86^	Working Memory	cTBS	Offline	Within	Accuracy	1	10	RC vs LC	Cerebellum	0,82	0,10	1,13	0,05	Y	NA	NA
^86^	Working Memory	cTBS	Offline	Within	RTs	1	10	RC vs LC	Cerebellum	0,13	0,08	0,17	0,04	Y	NA	NA
^87^	Working Memory	cTBS	Offline	Within	Accuracy	1	10	RC vs LC	Cerebellum	0,45	0,11	0,60	0,06	Y	NA	NA
^87^	Working Memory	cTBS	Offline	Within	RTs	2	13	RC vs LC	Cerebellum	0,48	0,09	0,67	0,05	y	NA	NA
^88^	Learning	LFrTMS	Offline	Between	RTs	1	36	RC	No TMS	0,27	0,27	0,27	0,27	Y	NA	NA
^89^	Learning	LFrTMS	Offline	Between	Accuracy	1	28	LC	Vertex	0,88	0,21	0,88	0,21	N	-0,88	-0,88

*TBS* theta-burst stimulation, *LFrTMS* low-frequency repetitive TMS, *tpTMS* triple-pulse TMS, *spTMS* single-pulse TMS, *HFrTMS* high-frequency repetitive TMS, *dpTMS * double-pulse TMS, *LC* left cerebellum, *RC* right cerebellum, *MC* medial cerebellum, *ES.5; V.5* effect size and variance with *r* = 0.5, *ES.75; V.75* effect size and variance with *r* = 0.75, *Exp* experiment, *N* sample numerosity, *CC* control condition, *Agg* aggregated data, *ES.5 direction; ES.75 direction* signed effect size. More information is available in the Supplementary Material.

$$\sqrt{{(SD}_{1}^{2}+{SD}_{2}^{2})-(2\times r\times {SD}_{1}\times {SD}_{2}})$$

That is, the mean difference is divided by the square root of the difference between the sum of the two squared standard deviations of the means (*SD*) and the multiplication among the two SDs and twice the correlation between the means (*r*).

For between-participants designs the effect size and variance calculation were performed using *R*^45^ and its package *compute.es*^46^ using the functions *mes* or *pes*. The calculation of Cohen’s *d* for within-participants was performed using a value of *r* = 0.75. This value was obtained by computing the correlation between measurements (pooling individual participants’ data) in four published papers investigating left cerebellar participation in social cognition^41^, spatial cognition^47^ and semantic memory^48,49^ and from one in preparation from our lab, investigating right cerebellar participation in semantic processing^50^. The computed correlations ranged from *r* = 0.64 to *r* = 0.95 (*Mean* = 0.78, *SD* = 0.10). To further control for the possible variability of this measure, we also computed Cohen’s *d* using a value of *r* = 0.5 and ran sensitivity analyses. All the Cohens’ *d* included were either used in their relative sign or transformed in their absolute value, due to the difficulty to estimate “negative” effects when employing brain stimulation techniques: we thus ran separated meta-analyses on signed and absolute effect-sizes.

For the studies employing a task explicitly used as a control task, only the data from the target task was used to calculate the effect size. We expected several studies to employ more than one task: a “target task”, which is thought to measure a specific function, and a “control task”, which is thought to measure a non-relevant (i.e., for the specific purpose of a certain study) function. When performing cerebellar TMS, the adoption of control tasks is particularly important to exclude non-specific effects. The absence of a cerebellar TMS effect in the control task is generally interpreted as evidence of the (possible) main effect of cerebellar TMS in the target task (thus excluding non-specific effects). In such cases, we consequently included only the data from the target task.

The effect size was then computed on the target task, measuring the difference between cerebellar TMS and a control TMS condition using the following rules (in hierarchical order; for more information about the control TMS condition, see Table 1): (i) when available sham, vertex stimulation or no TMS trials within cerebellar TMS session; (ii) if not available, the condition without TMS; (iii) stimulation of a control area; (iv) stimulation of another cerebellar area.

For the studies investigating pre vs. post cerebellar stimulation, if possible, we computed the effect size comparing post cerebellar TMS and post control condition (following the above-mentioned rules). If, within one study, more than one experiment was performed between cerebellar TMS and control conditions (i.e., including different samples of participants), we considered these experiments as independent ones.

For a certain number of studies, it was impossible to identify an effect more relevant than others (e.g., see:^42^, in which right cerebellar TMS was administered across five different tasks and no significant effects were found). In such cases (see Table 1), the effect sizes were aggregated using the R-package *MAd*^51^, with the function *agg*. This function simultaneously aggregates all the effect sizes implementing Borenstein procedure^52^ for aggregating dependent effect sizes. Please also note that data from different dependent variables were not aggregated, but kept separated.

When complete data were provided only graphically, effect sizes and variances were computed using the function *mes*, and the descriptive statistics were extracted from the figures using the WebPlotDigitizer software^53^.

### Meta-analyses

We performed two separate meta-analyses, one for each dependent variable (i.e., accuracy and RTs) on the absolute effect sizes. We also performed four distinct meta-regressions (i.e., two on accuracy and two on RTs as main dependent variables, respectively) to assess if the stimulation timing (online vs. offline) and the stimulation paradigm adopted (e.g., theta-burst stimulation, triple-pulse TMS, single-pulse TMS, etc.) moderated the observed effect. Two additional meta-regressions (i.e., one on each dependent variable) were performed to assess if the specific cognitive function (for a full list of cognitive functions see Table 1) moderated the observed effect.

Next, to investigate if the effect of cerebellar TMS was moderated by the site of stimulation (i.e., left vs. medial vs. right cerebellum), we first performed two meta-analyses (one for each dependent variable) excluding the studies which employed as control condition another cerebellar area, and then we performed two meta-regressions with the site of stimulation as moderator, again using the absolute effect sizes.

All the analyses were performed with restricted maximum-likelihood estimator method. The alpha for the *p*-values was set at = 0.01 (Bonferroni correction for multiple testing). The meta-analyses and meta-regressions performed, as well as the related plots, were computed using the R-package *metafor*^57^.

Heterogeneity was evaluated using the *Q*-test. In addition, we also report *I*^*2*^^54^, which provides the percentage of the total variability in the effect size estimation that could be attributed to heterogeneity among the true effect (heterogeneity is considered high if *I*^*2*^ > 75%^54^). To further investigate heterogeneity, we also computed the prediction intervals (*PI*) of the effect, which quantify the dispersion of effect. That is, 95% *PI* indicate the range of values that the effect size of a future study similar to those included should probably take.

Publication bias was evaluated using funnel plots, the trim-and-fill method^55^ and Egger’s test^56^. The trim-and-fill method provides an estimate of the number of studies missing from the meta-analysis due to the suppression of the most extreme results on one side (generally the left, i.e., non-significant results) of the plot. The Egger’s test examines if the funnel plot is asymmetric performing a regression of the effect size on the standard error weighted by the inverse variance, a significant *p* value indicates publication bias. To explore the robustness of the results, we performed a leave-one-out analysis: this procedure evaluates the robustness of the effect excluding one study at a time.

Finally, we performed sensitivity analyses performing the two main meta-analyses on accuracy and RTs including the effect sizes of the within-participants designs computed with *r* = 0.5.

Note that, as discussed before, we also we performed four additional meta-analyses (two on accuracy and two on RTs), this time using signed effect sizes (i.e., not transformed in absolute value; thus disentangling performance impairment from enhancement) and hence recomputing the two possible correlations between measurements. In this case, negative effect sizes index performance impairment, while positive effect sizes index performance enhancement. Yet, the analyses on signed effect sizes were performed only on studies comparing cerebellar vs. control condition different from the stimulation of another cerebellar site (in those comparing two cerebellar sites it is generally not possible to infer performance impairment or enhancement). Similarly, the studies in which there is no default performance impairment or enhancement (e.g., as in the case of pseudoneglect) were excluded from the analyses on signed effect size.

Additional information, including the plots of the meta-analyses performed using signed effect sizes as well as the tables with the number of the studies per condition of the non-significant meta-regressions is reported as Supplementary Material.

## Results

### Study selection

**Figure 1:**
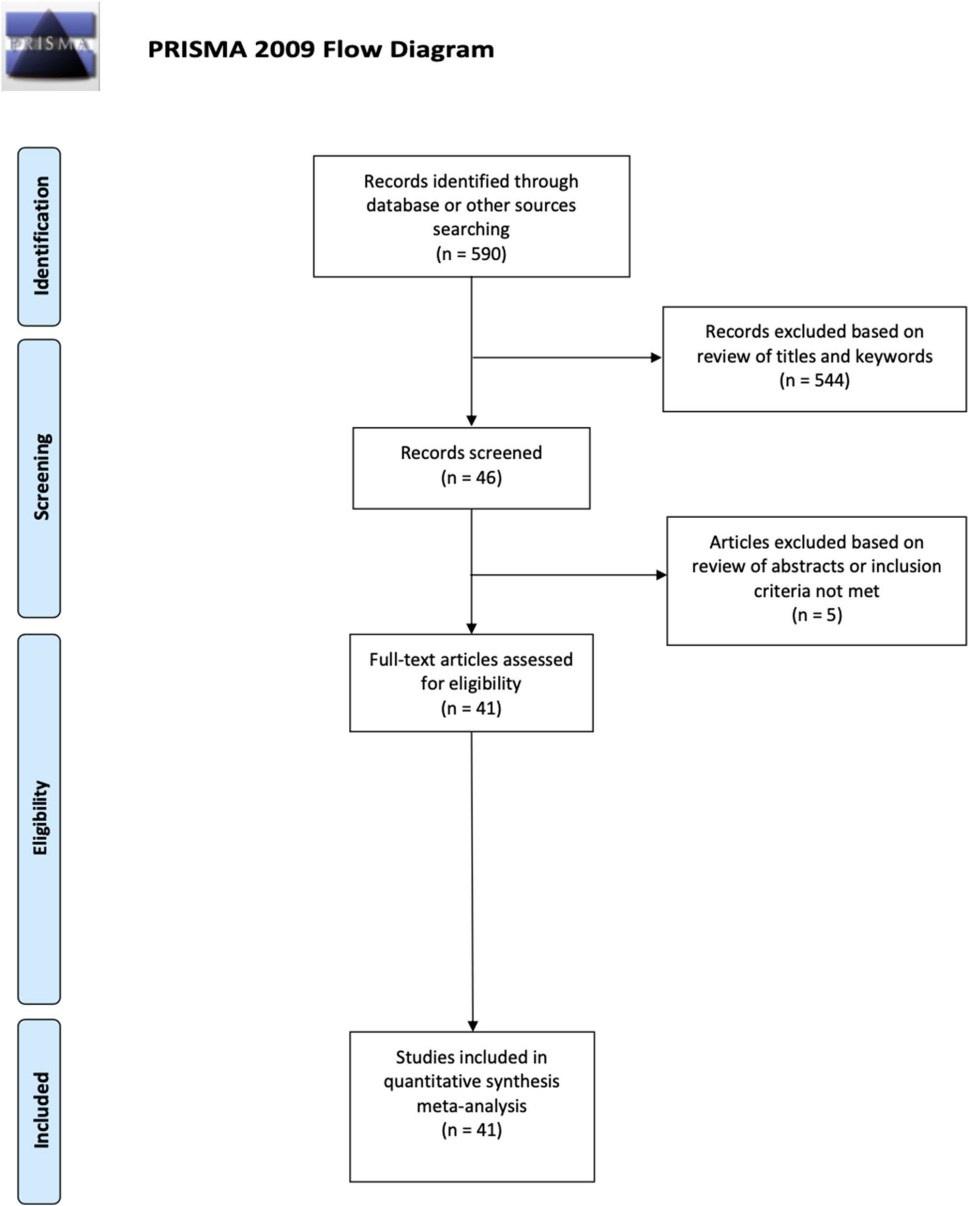
Flowchart illustrating study selection, review strategy and data extraction, retrieved from: http://prisma-statement.org/documents/PRISMA%202009%20flow%20diagram.pdf.

The literature search identified 590 articles (Fig. 1—PRISMA flowchart). Following the adoption of our selection criteria, a total of 41 studies were included in the present meta-analysis^37–42,47–49,58–89^). In total, because some studies reported both accuracy and RTs or performed more than one experiment, 85 effects were included, 44 on accuracy and 41 on RTs.

### Study characteristics

The characteristics of the included experiments are reported in Table 1. Studies were conducted between 2003 and 2020.

Of the 44 experiments included using accuracy as dependent variable, 11 were performed with a between-participants design, while 33 with a within-participants design. Of the 41 experiments included using RTs as dependent variable, 11 were performed with a between-participants design and 30 with a within-participants design.

For timing of stimulation, of the 44 experiments included using accuracy as dependent variable, 24 targeted the cerebellum while participants performed the task (i.e., online stimulation), while 20 targeted the cerebellum before the task or between two task sessions (i.e., offline stimulation). Of the 41 experiments included using RTs as dependent variable, 22 adopted online paradigms and 19 offline ones.

Concerning the stimulation paradigm, of the 44 experiments included using accuracy as dependent variable, 2 used double-pulse TMS (dpTMS), 3 high-frequency rTMS (HFrTMS), 7 low-frequency rTMS (LFrTMS), 3 single-pulse TMS (spTMS), 1 intermittent TBS (iTBS), 11 continuous TBS (cTBS), 17 triple-pulse TMS (tpTMS). Of the 41 experiments included using RTs as dependent variable, 2 used dpTMS, 2 HFrTMS, 5 LFrTMS, 3 spTMS, 14 cTBS and 15 tpTMS. Following^31^, we considered as HFrTMS those paradigms employing a TMS frequency > 1 Hz.

With regards to the specific cognitive function investigated, of the 44 experiments included using accuracy as dependent variable, 2 investigated attention, 1 episodic memory, 6 executive functions, 1 learning, 3 various sub-types of memory, 4 semantic memory, 9 social cognition, 5 spatial cognition, 6 timing and 7 working memory. Of the 41 experiments included using RTs as dependent variable, 6 investigated executive functions, 1 learning, 1 music, 11 semantic memory, 10 social cognition, 4 spatial cognition, 1 timing and 7 working memory. In particular, here the term *learning* refers to procedural learning, while with *various sub-types of memory* we target studies that employed tasks thought to measure more than one memory function (e.g., measuring both episodic and semantic memory^49^) or whose results have been aggregated across several memory functions (comprising episodic, semantic, working and short-term memory^42^). Finally, with *timing* we target studies assessing the representation and perception of time, thus not including musical processing.

### Accuracy

**Figure 2:**
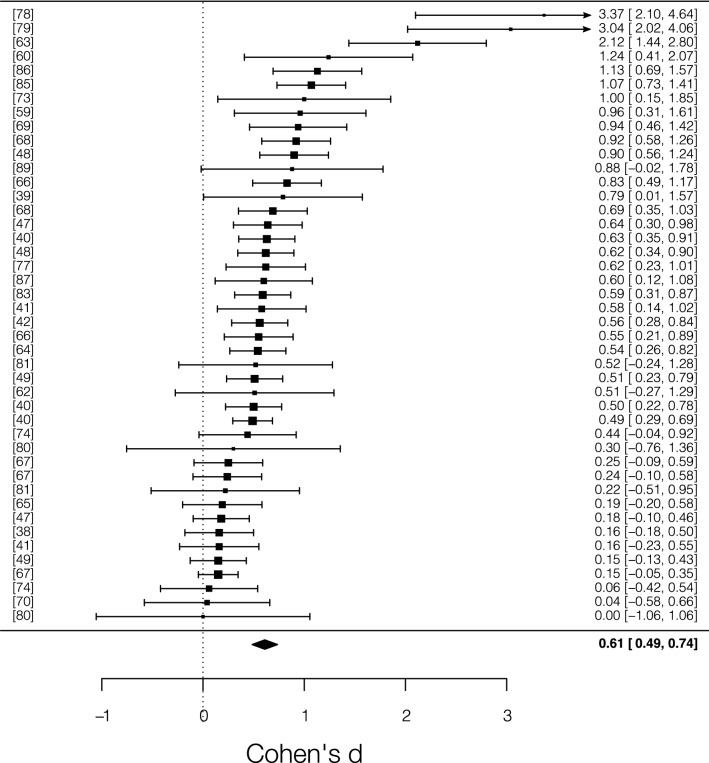
Forest plot of the studies that used accuracy as main dependent variable included in the meta-analysis. Each row corresponds to one experiment and the lines beside each square represent 95% confidence interval. The size of each square represents the weight of the study. The diamond at the bottom represents the cumulative effect size with 95% confidence interval. Higher positive values indicate higher behavioral modulation in the cerebellar TMS condition.

Random effects meta-analysis (*N* = 44) showed a medium mean effect size, *d* = 0.61 [95% *CI* = 0.48, 0.73; 95% *PI* = − 0.08, 1.30], *z* = 9.56, *p* < 0.0001, indicating that cerebellar TMS significantly affects participants’ accuracy compared to control conditions. Total heterogeneity was significant, *Q*_*T*_ = 157.68, *p* =  < 0001, *I*^*2*^ = 77%, suggesting moderate variance across the experiments included (Fig. 2).

**Figure 3:**
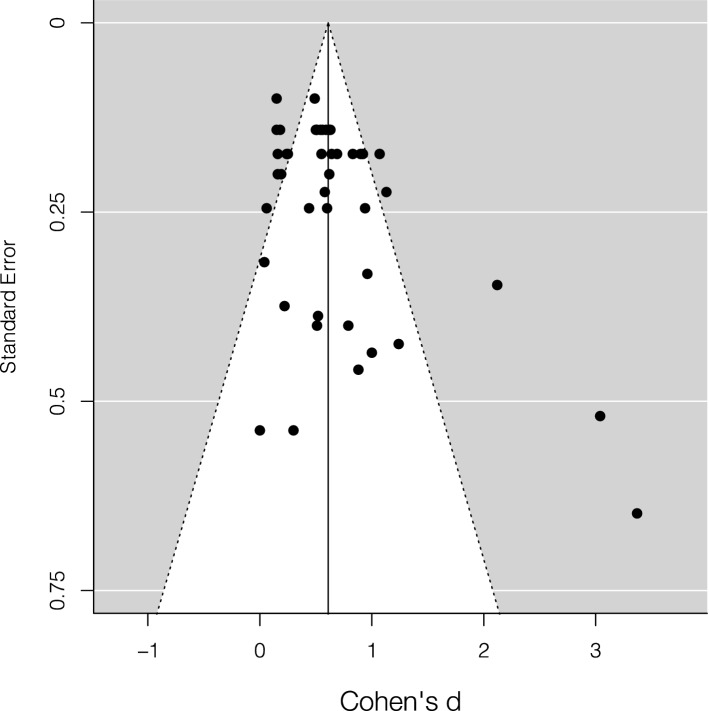
Funnel plot of the studies that used accuracy as main dependent variable included in the meta-analysis. Black dots represent the studies included. The vertical line represents the corrected effect size.

The leave-one-out analysis showed that the effect size was highly robust and ranged between 0.57 and 0.62 (*M* = 0.61, *SD* = 0.01). The trim and fill method did not add hypothetical missing studies on the left side of the funnel plot (Fig. 3). The Egger’s test was significant, *z* = 3.70, *p* = 0.0002, supporting the possibility of publication bias.

A first meta-regression did not show any moderation induced by stimulation timing (*N* = 44), *χ*^*2*^(1) = 3.50, *p* = 0.06. Heterogeneity remained significant, *Q*_*T*_ = 149.74, *p* < 0.0001, *I*^*2*^ = 76%.

A second meta-regression showed that stimulation paradigm moderated effects (*N* = 44), *χ*^*2*^(6) = 23.23, *p* = 0.0007. Heterogeneity remained significant, *Q*_*T*_ = 111.16, *p* < 0.0001, but *I*^*2*^ decreased, *I*^*2*^ = 67%. The decrease in heterogeneity suggests that the stimulation paradigm plays a role in determining the differences in the effects reported by the various studies (Table 2).

**Table 2:** Cohen’s *d* calculated using the stimulation paradigm as moderator.

	dpTMS	HFrTMS	LFrTMS	spTMS	iTBS	cTBS	tpTMS
Accuracy	**1.43**	**0.42**	**0.98**	0.32	0.19	**0.71**	**0.48**
	[0.92, 1.94]	[0.05, 0.79]	[0.66, 1.29]	[−0.03, 0.68]	[−0.47, 0.85]	[0.45, − 0.97]	[0.33, 0.64]
	*N* = 2	*N* = 3	*N* = 7	*N* = 3	*N* = 1	*N* = 11	*N* = 17

The effect for the study employing both TBS and HFrTMS (with aggregated effects) are not showed.

A third meta-regression did not show any moderation induced by the specific cognitive function investigated (*N* = 44), *χ*^*2*^(9) = 10.77, *p* = 0.29. Heterogeneity remained significant, *Q*_*T*_ = 127.97, *p* < 0.0001, *I*^*2*^ = 79%.

### Response times

**Figure 4:**
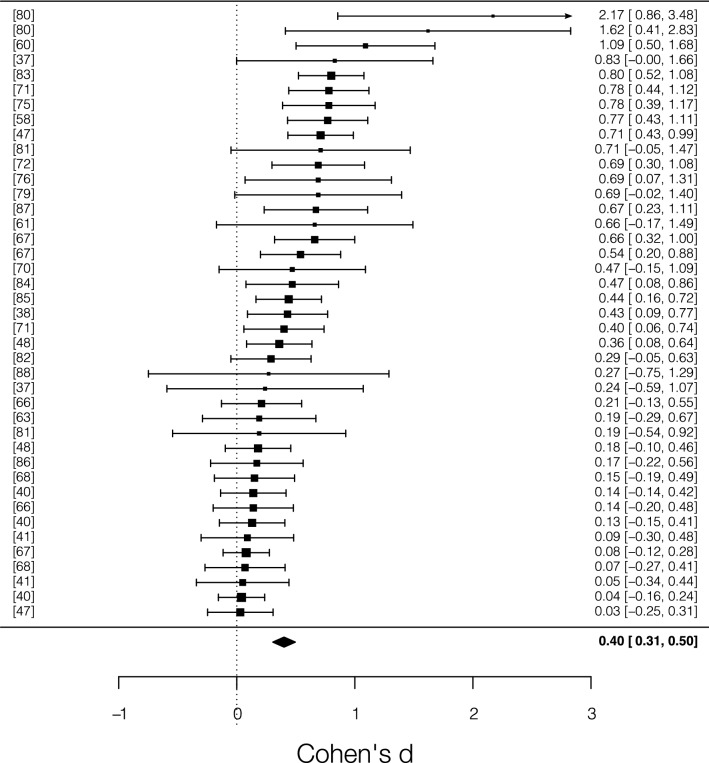
Forest plot of the studies that used RTs as main dependent variable included in the meta-analysis. Each row corresponds to one experiment and the lines beside each square represent 95% confidence interval. The size of each square represents the weight of the study. The diamond at the bottom represents the cumulative effect size with 95% confidence interval. Higher positive values indicate higher behavioral modulation in the cerebellar TMS condition.

The random effect meta-analysis (*N* = 41) showed a medium effect size, *d* = 0.40 [95% *CI* = 0.30, 0.49; 95% *PI* = -0.05, 0.85], *z* = 8.19, *p* < 0.0001, meaning that cerebellar TMS significantly affects participants’ RTs compared to control conditions. Total heterogeneity was significant and moderate, *Q*_*T*_ = 105.95, *p* < 0.0001, *I*^*2*^ = 60% (Fig. 4).

**Figure 5:**
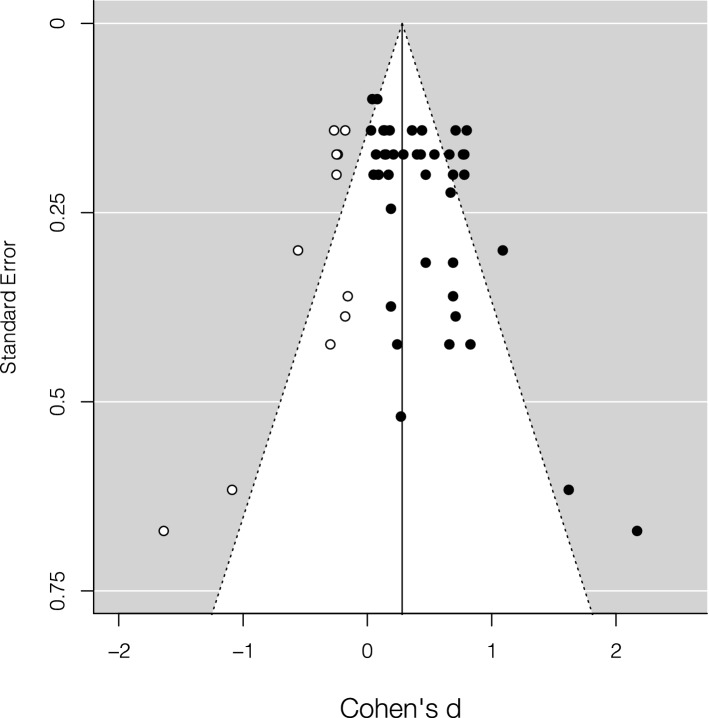
Funnel plot of the studies that used RTs as main dependent variable included in the meta-analysis. Black dots represent the studies included, while white dots represent hypothetical missing studies on the left side (estimated using the trim and fill method). The vertical line represents the corrected effect size.

The leave-one-out analysis showed that the effect size ranged between 0.38 and 0.41 (*M* = 0.40, *SD* = 0.008). The trim and fill method added 11 hypothetical missing studies on the left side of the funnel plot (Fig. 5). Adding these hypothetical studies, the effect size became smaller but still significant, *d* = 0.28 [95% *CI* = 0.17, 0.38], *z* = 5.11, *p* < 0.0001, and the heterogeneity remained significant, *Q*_*T*_ = 186.49, *p* < 0.0001, *I*^*2*^ = 73%. The Egger’s test was significant, *z* = 3.28, *p* = 0.001, supporting the possibility of publication bias.

A first meta-regression showed that stimulation timing (*N* = 41) moderated effects, *χ*^*2*^(1) = 17.25, *p* < 0.0001. Heterogeneity remained significant, *Q*_*T*_ = 71.45, *p* = 0.001, and *I*^*2*^ decreased, *I*^*2*^ = 44%, suggesting that the stimulation timing plays a role in determining the differences in the effects reported by the various studies. In particular, offline paradigms reported significantly higher effect sizes comparted with online ones, with both paradigms reporting cumulative effect sizes significantly different from zero (Table 3).

**Table 3:** Cohen’s *d* calculated using the stimulation timing as moderator.

	Online	Offline
RTs	**0.27**	**0.63**
	[0.17, 0.36]	[0.59, 0.77]
	*N* = 22	*N* = 19

A second meta-regression showed stimulation paradigm moderated effects (*N* = 41), *χ*^*2*^(5) = 26.44, *p* < 0.0001. Heterogeneity remained significant, *Q*_*T*_ = 57.22, *p* = 0.01, but *I*^*2*^ decreased, *I*^*2*^ = 37%. The decrease in heterogeneity suggests that the stimulation paradigm plays a role in determining the differences in the effects reported by the various studies (Table 4).

**Table 4:** Cohen’s *d* calculated using the stimulation paradigm as moderator.

	dpTMS	HFrTMS	LFrTMS	spTMS	cTBS	tpTMS
RTs	**0.35**	**0.58**	**0.73**	**0.38**	**0.59**	**0.20**
	[0.03, 0.67]	[0.23, 0.92]	[0.48, 0.99]	[0.15, 0.62]	[0.43, 0.75]	[0.09, 0.31]
	*N* = 2	*N* = 2	*N* = 5	*N* = 3	*N* = 14	*N* = 15

A third meta-regression did not show any moderation induced by the specific cognitive function investigated (*N* = 41), *χ*^*2*^(7) = 15.11, *p* = 0.03. Heterogeneity decreased slightly but remained significant, *Q*_*T*_ = 70.59, *p* = 0.0002, *I*^*2*^ = 53%.

### Cerebellar lateralization

#### Accuracy

The random effects meta-analysis (*N* = 35) showed a medium mean effect size, *d* = 0.54 [95% *CI* = 0.42, 0.66; 95% *PI* = − 0.03, 1.11], *z* = 8.79, *p* < 0.0001, indicating that cerebellar TMS significantly affects participants’ accuracy compared to control conditions. Total heterogeneity was significant, *Q*_*T*_ = 114.93, *p* ≤ 0001, *I*^*2*^ = 72%, suggesting moderate variance across the experiments included.

The leave-one-out analysis showed that the effect size was highly robust and ranged between 0.50 and 0.55 (*M* = 0.54, *SD* = 0.01). The trim and fill method did not add hypothetical missing studies on the left side of the funnel plot. The Egger’s test was significant, *z* = 2.17, *p* = 0.02, supporting the possibility of publication bias.

A meta-regression did not show any moderation induced by the stimulation site (*N* = 35), *χ*^*2*^(2) = 1.37, *p* = 0.50. Heterogeneity remained significant, *Q*_*T*_ = 108.17, *p* < 0.0001, *I*^*2*^ = 71%.

#### Response times

The random effects meta-analysis (*N* = 34) showed a medium mean effect size, *d* = 0.34 [95% *CI* = 0.25, 0.44; 95% *PI* = − 0.04, 0.73], *z* = 7.12, *p* < 0.0001, indicating that cerebellar TMS significantly affects participants’ accuracy compared to control conditions. Total heterogeneity was significant, *Q*_*T*_ = 76.82, *p* ≤ 0001, *I*^*2*^ = 54%, suggesting moderate variance across the experiments included.

The leave-one-out analysis showed that the effect size was highly robust and ranged between 0.32 and 0.36 (*M* = 0.34, *SD* = 0.009). The trim and fill method added 4 hypothetical missing studies on the left side of the funnel plot. Adding these hypothetical studies, the effect size became smaller but still significant, *d* = 0.30 [95% *CI* = 0.21, 0.40], *z* = 6.26, *p* < 0.0001, and the heterogeneity remained significant, *Q*_*T*_ = 95.50, *p* < 0.0001, *I*^*2*^ = 56%. The Egger’s test was significant, *z* = 3.23, *p* = 0.001, supporting the possibility of publication bias.

A meta-regression did not show any moderation induced by the stimulation site (*N* = 34), *χ*^*2*^(2) = 6.19, *p* = 0.04. Heterogeneity decreased slightly but remained significant, *Q*_*T*_ = 61.86, *p* < 0.0001, *I*^*2*^ = 46%.

### Sensitivity analyses

The sensitivity analyses performed are reported in Table 5. Besides the two meta-analyses reported above, with *r* = 0.75 for correlation between measures in within-participants designs, we also performed two meta-analyses with *r* = 0.5. The meta-analyses performed with *r* = 0.5 show lower cumulative effect sizes (*d* = 0.41 for accuracy; *d* = 0.27 for RTs), but both are still significant. The two meta-analysis performed with *r* = 0.5 report also substantially different indexes in all the other measures assessed. In particular, heterogeneity is reduced for *r* = 0.5, but funnel plot asymmetry is still problematic, with the trim and fill method estimating a large number of studies missing (7 for accuracy; 11 for RTs). Egger’s test is also significant.

**Table 5:** Results of the meta-analyses performed with the effect sizes of the within-participants studies included computed using *r* = 0.75 or *r* = 0.5.

Dependent variable	Corr	Effect size	*Q-*test	Higgins’*I*^*2*^	Trim and fill	Egger’s test
Accuracy	*r* = 0.75	**0.60** [*CI* = .47, .73; *PI* = -.09, 1.30]	***p* < .0001**	77%	0	***p* = .0002**
	*r* = 0.5	**0.41** [*CI* = 0.33, 0.49; *PI* = 0.22, 0.60]	***p* = .005**	11%	7	***p* < .0001**
RTs	*r* = 0.75	**0.40** [*CI* = 0.30, 0.49; *PI* = −0.05, 0.85]	***p* < .0001**	60%	11	***p* = .001**
	*r* = 0.5	**0.27** [*CI* = 0.19, 0.35; *PI* = 0.16, 0.38]	*p* = .27	3%	11	***p* < .0001**

### Signed effect sizes

The results of the meta-analyses on signed effect sizes are reported in Table 6. As above, we performed on accuracy and RTs two meta-analyses with *r* = 0.75 and *r* = 0.5 for correlation between measures in within-participants designs. In this case we used the signed effect sizes, with negative values indicating performance impairment and positive values indicating performance enhancement. For accuracy, both meta-analyses reported a negative cumulative effect size, indicating that generally the studies included reported performance impairment. Conversely, for RTs, both meta-analyses reported non-significant cumulative effect sizes, likely indicating that positive and negative effects countered each other. As above, with higher correlation between measures, heterogeneity was higher and publication bias followed the same pattern (but was less evident for accuracy).

**Table 6:** Results of the meta-analyses performed on signed effect sizes with the effect sizes of the within-participants studies included computed using *r* = 0.75 or *r* = 0.5.

Dependent variable	Corr	Signed effect size	*Q-*test	Higgins’ *I*^*2*^	Trim and fill	Egger’s test
Accuracy	*r* = 0.75	**−0.45** [*CI* = −0.62, −0.27; *PI* = −1.29, 0.39]	***p* < .0001**	84%	0	*p* = .67
	*r* = 0.5	**−0.32** [*CI* = −0.45, −0.20; *PI* = −0.76, 0.10]	***p* = .007**	42%	3	*p* = .34
RTs	*r* = 0.75	−0.13 [*CI* = −0.30, 0.02; *PI* = −0.92, 0.65]	***p* < .0001**	83%	3	***p* = .02**
	*r* = 0.5	−0.09 [*CI* = −0.21, 0.02; *PI* = −0.51, 0.31]	***p* = .0009**	41%	4	***p* = .006**

## Discussion

Because of the main propriety of magnetic stimulation, which allows to infer causal relationships between a targeted brain area and a specific cognitive function, as well as the growing interest around cerebellar involvement in non-motor functions, the number of studies targeting the cerebellum using TMS has largely increased in the past few years. In particular, while several studies showed that the cerebellum is clearly involved in motor coordination and adaptation^19–24^, the findings about cerebellar involvement in non-motor functions were qualitatively more variable. In the present meta-analysis, we thus aimed at quantifying the effects of TMS applied over the cerebellum on non-motor functions for both accuracy and RTs. Our results showed that TMS is a reliable technique for investigating cerebellar participation in cognitive processes. TMS administered over the cerebellum was indeed found to successfully modulate cognitive performance (either in terms of cognitive impairment or enhancement), affecting accuracy and RTs. The cumulative effects calculated were robust and heterogeneity was partly accounted by the moderators added in meta-regressions for both accuracy and RTs data. Critically, the effects of TMS were significant not only when considering a strong correlation among measurements (*r* = 0.75) for within-participants designs, but also for a moderate correlational value (*r* = 0.5).

In this study, we further investigated whether other potentially crucial variables, namely stimulation timing, stimulation paradigm or the specific cognitive function investigated, could moderate the observed effects. We found that stimulation timing moderated the observed effects on RTs only (and not on accuracy), with cumulative effect sizes being significantly higher for offline compared with online paradigms. For both dependent variables, the stimulation paradigm moderated the observed effects, suggesting that the various stimulation paradigms do play a role in determining the effect of cerebellar TMS. These results indicate that certain TMS paradigms can be more reliable than others when investigating cerebellar functions. Conversely, for both dependent variables, the specific cognitive function investigated did not moderate the observed effects, indicating similar effects across the various functions at hand.

Finally, we also investigated the possible effect of lateralization (i.e., whether cerebellar TMS effects depend on the stimulation site being left vs. medial vs. right), including this variable as a moderator in a meta-regression. Across both accuracy and RTs, we found that TMS site did not moderate the observed effects, suggesting similar effects across the three cerebellar sites tested (left vs. medial vs. right cerebellum). Unfortunately, a deeper relationship between TMS site and the specific cognitive function investigated could not be handled here due to the low numerosity within each group, but would likely modulate the observed effects, since certain cognitive functions appear to be lateralized in the cerebellum^90,91^. Indeed, a large number of studies focusing on social cognition specifically targeted the left cerebellum only^67,68^, in line with neuroimaging evidence showing left cerebellar activations during social tasks^92,93^. Similarly, studies focusing on semantic and linguistic processing, as well as on verbal working memory, mainly targeted the right cerebellum^49,60^. The laterality of these cognitive functions reflects the fact that cerebro-cerebellar interactions and cerebro-cerebellar connections are crossed^94^. Therefore, because many studies targeted only the left or the right cerebellum as a function of the specific cognitive process tested, we could not address the interaction between laterality and cognitive function directly in our meta-analysis. Indeed, only a small number of available studies directly focused on cerebellar asymmetries (e.g.,^71,79^), with this topic being particularly promising for future research in order to distinguish between left vs. medial vs. right cerebellar involvement in cognitive processing.

Another critical point is related to the effect size differences between the two dependent variables considered (i.e., RTs and accuracy), which may seem surprising, as from an experimental point of view both these measures quantify participants’ performance and are generally highly related (e.g., as in the case of speed-accuracy tradeoff). Moreover, because of cerebellar involvement in event timing^95,96^, one may have expected higher effect sizes for RTs than for accuracy. However, the specific stimulation paradigm adopted, the specific stimulation timing (i.e., offline vs. online stimulation) and the specific task adopted can all play a critical role on the observed behavior. That is, the pattern found may be affected by the interaction among these variables as well as by the involvement of other cerebral areas in the specific function tested. This interpretation is consistent with previous evidence showing that TMS effects depend on various factors such as stimulation intensity, brain state and timing^97^, and that TMS does not simply cause a generalized “virtual lesion”^98^.

We believe that our findings contribute to the debate on the role of the cerebellum in cognitive functions from both methodological and theoretical points of view. Firstly, our findings provide cumulative information quantifying the effect of the various TMS paradigms on cerebellar functions, as well as the effect of the various TMS timing and sites on cerebellar functions. Secondly, our meta-analysis supports cerebellar involvement in non-motor processing, indicating that the cerebellum does participate in cognitive processing and that this involvement is not moderated by the specific function investigated. This evidence further supports theories regarding cerebellar involvement in cognitive processing^7,99^. In addition to this, the cumulative effect sizes computed may have a direct application, allowing researchers to use them when estimating the minimum sample size needed to observe the hypothesized effect in future studies.

Regarding the effects of TMS on cerebellar cortex, it has been suggested that TMS directly modulates inhibitory activity of Purkinje cells placed in cerebellar cortex, thus affecting the activity of the cerebral cortex via the thalamus^100^. Purkinje cells activity can modulate size, speed, and timing of movements^101^ and it has been shown that when posterior cerebellar areas are targeted with TMS, the activity of other brain areas (e.g., deep nuclei, prefrontal areas, thalamus, etc.^36,65,102,103^) is modulated. Critically, the effects of cerebellar TMS on the activity of other brain areas as measured by motor-evoked potential (MEP) are frequency dependent. It has indeed been shown that LFrTMS and iTBS enhance MEPs^104,105^, while cTBS exerts the opposite effect^106^. Investigating such effects in future studies may be critical from a clinical point of view, particularly for the use of TMS in rehabilitation protocols for neuropsychiatric disorders^107,108^.

In interpreting our findings, three main limitations should be considered; namely, the potential publication bias, the fact that we mainly focused on absolute effect sizes and the level of heterogeneity. First, evidence for publication bias was particularly strong in sensitivity analyses considering a smaller correlation for within-participant designs. Various authors pointed to the need of interpreting null TMS results and making such findings available to the scientific community^109^, possibly providing more detailed evidence for the involvement of certain brain areas in certain functions. Second, concerning the issue of heterogeneity, our findings were strictly dependent on the correlation used for effect sizes estimation. This pattern of results must be interpreted by considering that almost none of the within-participants study included reported the correlation between measurements, leading to imprecise effect size computation. Nevertheless, for both dependent variables we found that prediction intervals were large and their lower bound negative, indicating that although cerebellar TMS is effective on average in modulating human cognitive performance, heterogeneity is also high. Given cerebellar anatomo-physiological characteristics, we believe that the high heterogeneity is likely related to differences in timing, intensity and TMS procedures adopted by the studies included, some of which could not be analyzed here. In addition, using a smaller correlation coefficient between the two measurements, heterogeneity was more contained, emphasizing the importance of reporting the correlation in within-participant designs. Finally, in our main meta-analyses we focused on absolute values of effect sizes, as we were interested in probing whether TMS generally modulates cognitive performance, regardless of the specific direction of the considered effect (i.e., whether TMS induces cognitive impairment or enhancement). In addition to this, defying a priori whether a certain TMS paradigm would result in impairment or enhancement may be problematic and rather unrealistic. Our approach may have therefore increased the likelihood of observing significant effects, affecting in turn the publication bias and inducing consequently an asymmetric funnel plot. Yet, we also note that when we computed signed effect sizes, thus accounting for the specific direction of modulation (i.e., impairment or enhancement), we still found that TMS administered over the cerebellum was able to successfully modulate cognitive performance, showing that cerebellar TMS typically results in cognitive impairment in terms of accuracy.

In conclusion, the present meta-analysis indicates that TMS is effective in modulating cerebellar activity. These results therefore substantiate the well-established dysmetria of thought hypothesis^7^, corroborating the idea that the cerebellum is involved in non-motor, cognitive functions.

## Supplementary Information

Supplementary Information.

Supplementary Table S1.
