# Supplementary material for: Probing cerebellar involvement in cognition through a meta-analysis of TMS evidence
Source: Sci Rep. 2021 Jul 20;11:14777. doi: 10.1038/s41598-021-94051-5 (PMC8292349; doi:10.1038/s41598-021-94051-5)
Supplement: Supplementary file 1 — Supplementary Information. [file 41598_2021_94051_MOESM1_ESM.pdf]

Supplementary Material

Probing cerebellar involvement in cognition through a meta-analysis of TMS evidence

Daniele Gatti[1]*, Luca Rinaldi[1], Ioana Cristea[1], & Tomaso Vecchi[1,2]

[1] *Department of Brain and Behavioral Sciences, University of Pavia, Pavia, Italy*

[2] *Cognitive Psychology Unit, IRCCS Mondino Foundation, Pavia, Italy*

* Corresponding author:

Daniele Gatti, Department of Brain and Behavioural Science, University of Pavia, Piazza Botta 6, 27100 Pavia, Italy. *e-mail address*: daniele.gatti@unipv.it

Additional information

We report below the tables with the number of the studies per condition of the non-significant meta-regressions included in the manuscript.

Online vs. Offline

Table S2. Study numerosity of the first meta-regression on accuracy.

	**Online**	**Offline**
Timing	*N* = 24	*N* = 20

Cognitive functions

Table S3. Study numerosity of the third meta-regression on accuracy.

	**Attention**	**Episodic memory**	**Executive functions**	**Learning**	**Memory**	**Semantic memory**	**Social cognition**	**Spatial cognition**	**Timing**	**Working memory**
Cognitive Function	*N* = 2	*N* = 1	*N* = 6	*N* = 1	*N* = 3	*N* = 4	*N* = 9	*N* = 5	*N* = 6	*N* = 7

Table S4. Study numerosity of the third meta-regression on RTs.

	**Executive functions**	**Learning**	**Music**	**Semantic memory**	**Social cognition**	**Spatial cognition**	**Timing**	**Working memory**
Cognitive Function	*N* = 6	*N* = 1	*N* = 1	*N* = 11	*N* = 10	*N* = 4	*N* = 1	*N* = 7

Cerebellar lateralization

Table S5. Study numerosity of the meta-regression on accuracy and lateralization.

	**Left**	**Middle**	**Right**
Cerebellar site	*N* = 16	*N* = 5	*N* = 14

Table S6. Study numerosity of the meta-regression on RTs and lateralization.

	**Left**	**Middle**	**Right**
Cerebellar site	*N* = 14	*N* = 6	*N* = 14

Signed effect sizes

The forest plots of the meta-analyses on signed effect sizes with $r = .75$ are reported in Figure S1 (accuracy) and Figure S2 (RTs), respectively.

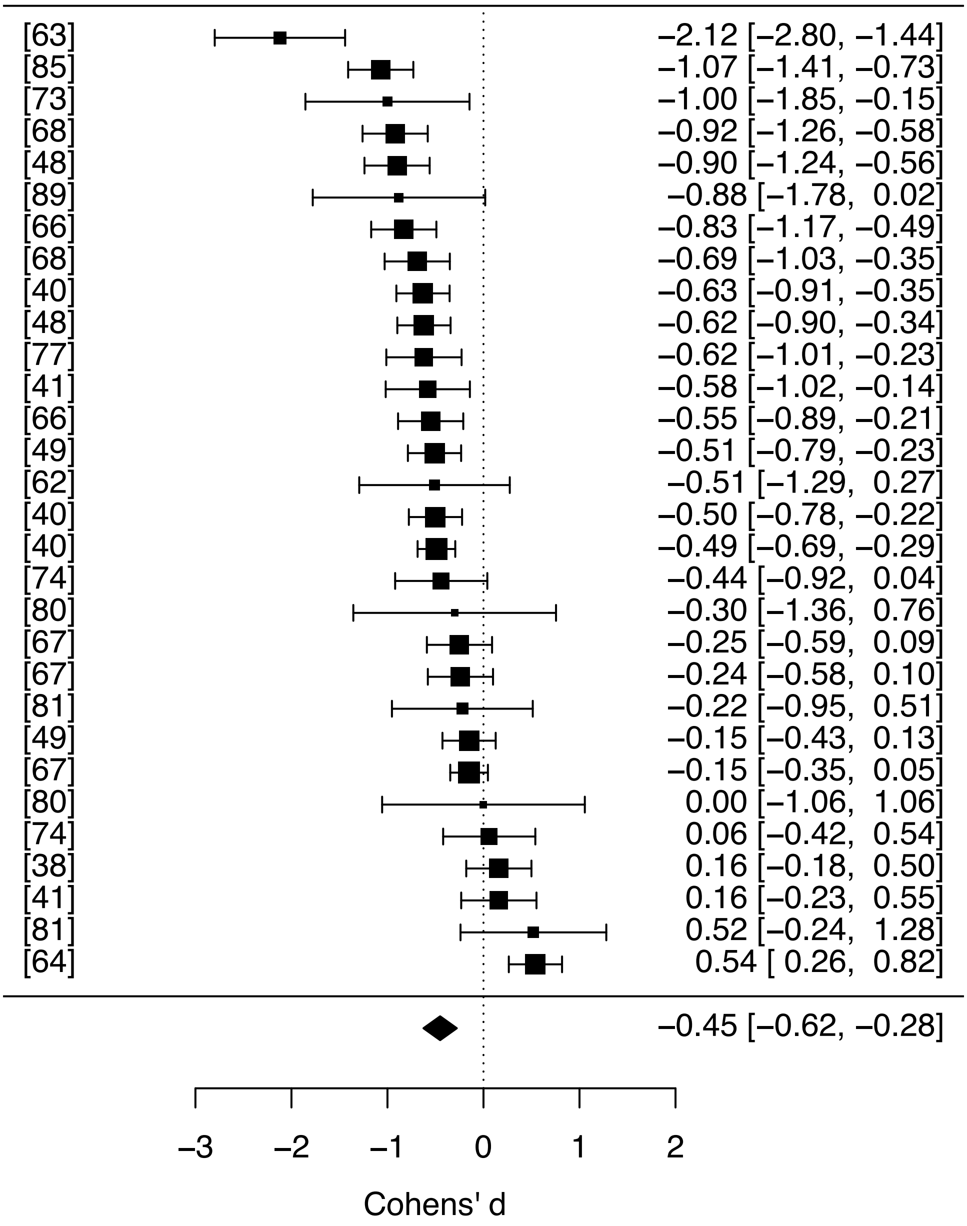

Figure S1. Forest plot of the studies that used accuracy as main dependent variable included in the meta-analysis with signed effect sizes. Each row corresponds to one experiment and the lines beside each square represent 95% confidence interval. The size of each square represents the weight of the study. The diamond at the bottom represents the cumulative effect size with 95% confidence interval. Negative values indicate performance impairment, positive values indicate performance enhancement.

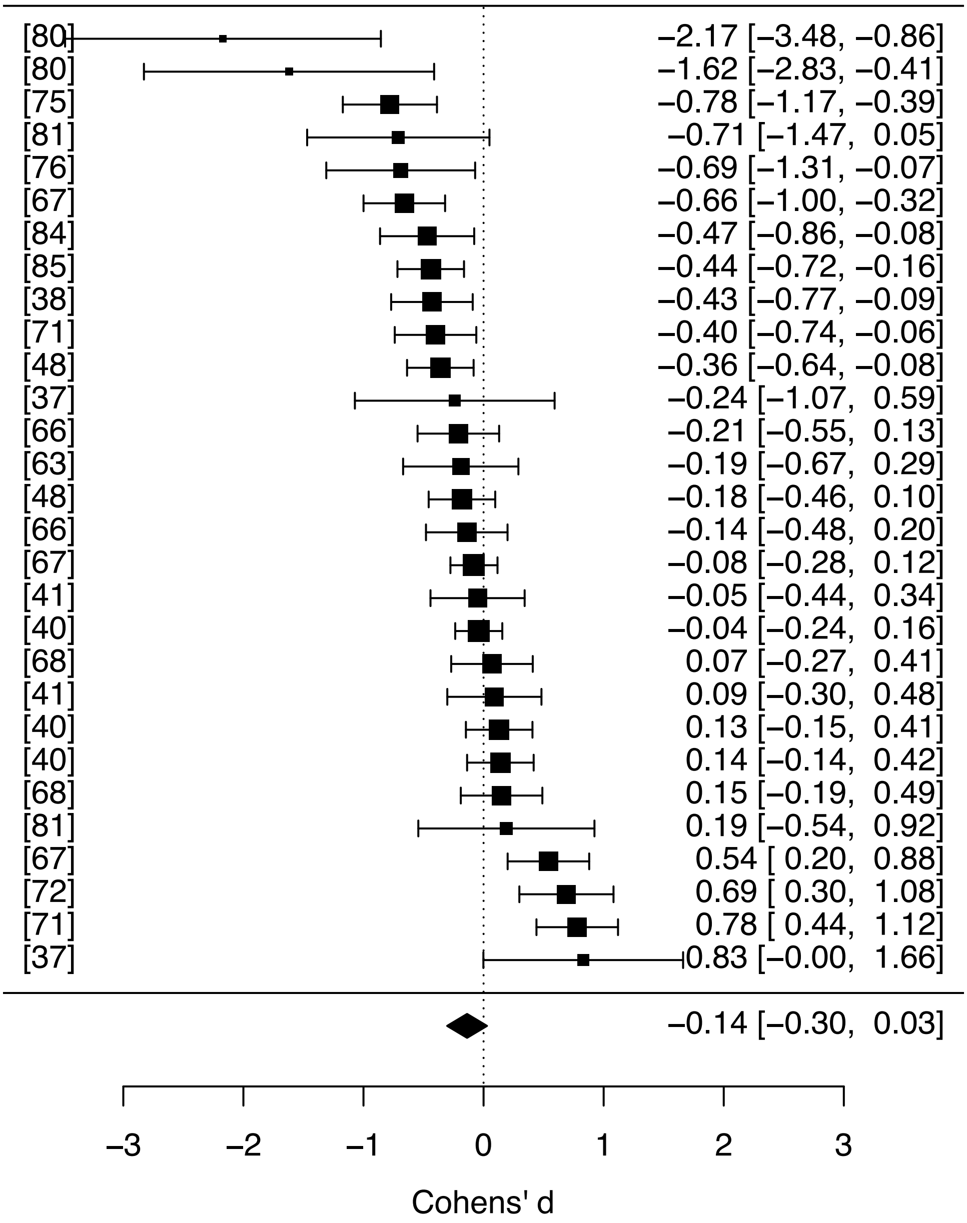

Figure S2. Forest plot of the studies that used RTs as main dependent variable included in the meta-analysis with signed effect sizes. Each row corresponds to one experiment and the lines beside each square represent 95% confidence interval. The size of each square represents the weight of the study. The diamond at the bottom represents the cumulative effect size with 95% confidence interval. Negative values indicate performance impairment, positive values indicate performance enhancement.
